# Alport syndrome cold cases: Missing mutations identified by exome sequencing and functional analysis

**DOI:** 10.1371/journal.pone.0178630

**Published:** 2017-06-01

**Authors:** Chiara Chiereghin, Michela Robusto, Antonio Mastrangelo, Pierangela Castorina, Giovanni Montini, Marisa Giani, Stefano Duga, Rosanna Asselta, Giulia Soldà

**Affiliations:** 1 Department of Biomedical Sciences, Humanitas University, Rozzano, Milan, Italy; 2 Humanitas Clinical and Research Center, Rozzano, Milan, Italy; 3 UOC Nefrologia Pediatrica, Fondazione IRCCS Cà Granda Ospedale Maggiore Policlinico, Milan, Italy; 4 UO Audiologia, Fondazione IRCCS Cà Granda Ospedale Maggiore Policlinico, Milan, Italy; 5 Dipartimento di Scienze Cliniche e di Comunità, Università degli Studi di Milano, Milan, Italy; International Centre for Genetic Engineering and Biotechnology, ITALY

## Abstract

Alport syndrome (AS) is an inherited progressive renal disease caused by mutations in *COL4A3*, *COL4A4*, and *COL4A5* genes. Despite simultaneous screening of these genes being widely available, mutation detection still remains incomplete in a non-marginal portion of patients. Here, we applied whole-exome sequencing (WES) in 3 Italian families negative after candidate-gene analyses. In Family 1, we identified a novel heterozygous intronic variant (c.2245-40A>G) -outside the conventionally screened candidate region for diagnosis- potentially disrupting *COL4A5* exon29 splicing. Using a minigene-based approach in HEK293 cells we demonstrated that this variant abolishes exon29 branch site, causing exon skipping. Moreover, skewed X-inactivation of the c.2245-40A>G allele correlated with disease severity in heterozygous females. In Family 2, WES highlighted a novel *COL4A5* hemizygous missense mutation (p.Gly491Asp), which segregates with the phenotype and impacts on a highly-conserved residue. Finally, in Family 3, we detected a homozygous 24-bp in-frame deletion in *COL4A3* exon1 (NM_000091.4:c.30_53del:p.Val11_Leu18del or c.40_63del24:p.Leu14_Leu21del), which is ambiguously annotated in databases, although it corresponds to a recurrent AS mutation. Functional analyses showed that this deletion disrupts COL4A3 signal peptide, possibly altering protein secretion. In conclusion, WES -together with functional studies- was fundamental for molecular diagnosis in 3 AS families, highlighting pathogenic variants that escaped previous screenings.

## Introduction

Alport syndrome (AS) is a rare inherited disease of the glomerular basement membrane (GBM) with a prevalence of 1–10:50,000 live births in different populations [1] and characterized by hematuria, with first onset usually in childhood, proteinuria, and progressive renal failure. Extrarenal signs might also be present, including sensorineural hearing loss and ocular anomalies, such as anterior lenticonus, cataract, and maculopathy [2]. AS is caused by mutations in the *COL4A3*, *COL4A4*, and *COL4A5* genes, encoding the α3, α4, and α5 chains of collagen type IV, a major structural component of the GBM, as well as of the basement membranes in the cochlea and eye. Kashtan and colleagues [2] recently reported that ~65% of AS cases are X-linked (MIM#301050, *COL4A5* mutations), 20% are autosomal dominant (MIM#104200, *COL4A3* or *COL4A4* heterozygous mutations), and the remaining 15% are autosomal recessive (MIM#203780, biallelic mutations in *COL4A3* or *COL4A4*). Previously, autosomally dominant inherited forms were considered rare [1]. In a small number of cases, evidence for digenic inheritance of AS has also been reported [3].

Some heterozygous mutations in *COL4A3* and *COL4A4* genes can cause a milder phenotype, defined as thin basement membrane nephropathy (TBMN; MIM#141200, or benign familial hematuria), which is characterized by persistent microscopic hematuria although rarely combined with progressive proteinuria and end-stage renal disease [4].

AS shows high inter- and intra-familial phenotypic variability, as well as high allelic heterogeneity [5]. Indeed, >900 different mutations have been collectively reported in the three collagen IV genes (Human Gene Mutation Database, HGMD: http://hgmd.cf.ac.uk, last accessed October 2016). In addition, these genes are large -comprising 52, 48, and 51 coding exons for *COL4A3*, *COL4A4*, and *COL4A5*, respectively- thus hindering comprehensive genetic screenings in large patient series. To further complicate the scenery, genetic variations in modifier genes, such as *NPHS2* (encoding podocin), may modify disease severity in patients who have at least one mutation in a type IV collagen gene [6].

In recent years, the introduction of next-generation sequencing (NGS) has made possible the time- and cost-effective analysis of all three AS genes in a single step [7,8]. Despite these technological advancements, the total mutation detection rate ranged from 55 to 80%, meaning that at least a fifth of the patients still remains without a molecular diagnosis [8,9]. Unequivocal -and possibly early- molecular diagnosis is extremely important for prognostic assessment and genetic counseling.

Reasons for missing pathogenic mutations by NGS can be multiple. For example, GC-rich exons may fail capture or amplification and subsequent sequencing. Moreover, the presence of homopolymeric repeats might be a specific issue when using certain NGS platforms [10]. In addition, detection of small insertion-deletions (indels) and gene rearrangements is known to be less accurate than single nucleotide variants [8]. Finally, deep-intronic and promoter mutations can be missed by routine screening as well as standard NGS data analysis, since they are mostly focused on coding exons and splice sites.

Recently, whole-exome sequencing (WES) has been proposed as an alternative approach in cases negative for gene-specific screenings [8,9]. The existence of additional so-far unknown genes for Alport and Alport-like disease has also been suggested [9].

Here, we applied WES to identify the genetic basis of AS in three Italian families with clear clinical evidence but no molecular diagnosis, despite having been subjected to extensive analyses -including targeted NGS resequencing of all three collagen genes- over the last 10 years (S1 Table).

## Materials and methods

### Subjects

This study was approved by the Ethical Committee of the Fondazione IRCCS Cà Granda Ospedale Maggiore Policlinico of Milan and performed according to the Declaration of Helsinki. Signed informed consent was obtained from all participants and from parents of subjects younger than 18 years.

Genomic DNA was extracted from peripheral blood using an automated DNA extractor Maxwell 16 system (Promega, Madison, WI), and quantified on a Nanodrop ND-1000 spectrophotometer (NanoDrop Technologies, Wilmington, DE).

Three Italian families were included in this study. Diagnosis of AS was primarily based on renal biopsy, which showed pathognomonic ultrastructural abnormalities in the GMB, such as thickening, splitting, and reticulation, as well as on at least two other criteria, including: i) family history of hematuria, chronic kidney disease or AS; ii) SHL; and/or iii) specific ocular anomalies (Table 1).

**Table 1:** Patient characteristics.

Patient	Gender	Age	Family history	Proteinuria/creatinuria	GBM changes	Renal function	Hearing loss	Ocular lesions
**P1**	F	47	AS	0.4	Thinning, thickening	Normal	N	N
**P2**	M	20	HE, SHL	>2	Thinning, thickening, splitting	CKD	Y	N
**P3**	F	25	HE	>1	Thinning, thickening, splitting	Normal	Y	Y

AS: Alport syndrome; HE: hematuria, SHL: Sensorineural Hearing Loss; CKD: Chronic Kidney Disease; F: female; M: male; N: no; Y: yes.

The probands from the three families were previously subjected to several genetic screenings, as reported in S1 Table.

### Whole-exome sequencing

**Fig 1:**
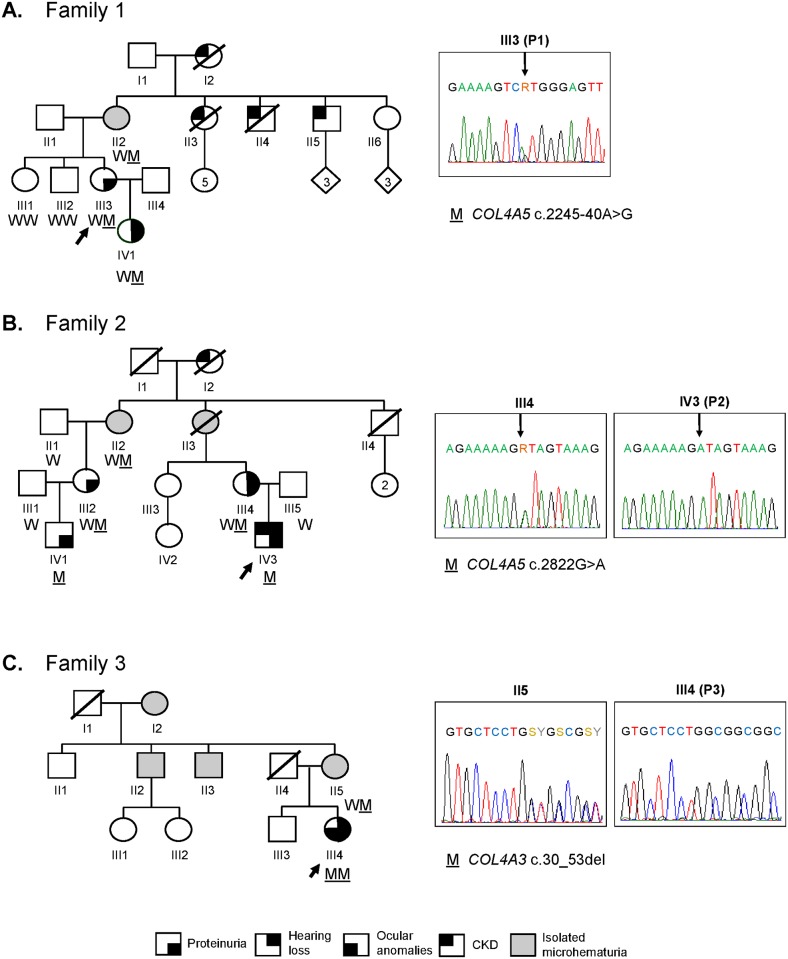
Identification of candidate *COL4A5* and *COL4A3* variants segregating with Alport syndrome in three Italian families. Pedigrees of Family 1 (A, X-linked), 2 (B, X-linked), and 3 (C, autosomal recessive) showing the segregation of the identified variants with AS. Individuals analyzed by WES are pointed by an arrow. The genotype of available individuals from each family is indicated below the corresponding symbols and illustrative electropherograms are shown on the right. M, mutant; W, wild type; R, A or G; S, G or C; Y, C or T; CKD, chronic kidney disease.

WES was performed on the three probands (P1-P3, Fig 1). Sequencing libraries were prepared starting from 50 ng of genomic DNA using the Nextera Rapid Capture Exome Enrichment kit (Illumina, San Diego, CA), following the manufacturer’s instructions, and run as 150-bp paired-end reads on a NextSeq500 (Illumina).

Reads were aligned to the human reference genome (hg19, GRCh37 build) using the Burrows-Wheeler Aligner (BWA) version 0.7.7 program [11], duplicates were marked with Picard v.1.79 and genotypes at variant positions (both single nucleotide variants and indels) were called using GATK v.1.6–23 [12].

Variant annotation and prioritization is detailed in Supporting Methods. Variant analysis primarily focused on *COL4A3*, *COL4A4*, and *COL4A5*. Both exonic and intronic variants were evaluated.

### Sanger sequencing

*COL4A5* exons 29 (including flanking intronic sequences) and 33, as well as *COL4A3* exon 1 were PCR amplified using sets of primers designed on the basis of the known genomic sequence of the gene (GenBank accession numbers NM_000495.4 and NM_000091.4). PCRs were performed on 10–20 ng of genomic DNA, following standard procedures. Primer sequences and specific PCR conditions are available on request. Direct sequencing of amplified fragments was performed on both strands with the BigDye Terminator Cycle Sequencing Ready Reaction Kit v.1.1 and an automated ABI-3500DX DNA sequencer (Applied Biosystems, Foster City, CA). The Variant Reporter software (Applied Biosystems) was used for variant detection.

### *In-silico* analyses

Branch-point sequence analysis of the wild-type and mutant *COL4A5* intron 28-exon 29 boundary was performed using the Human Splicing Finder software (http://www.umd.be/HSF3/, last accessed October 2016).

Signal-peptide prediction of wild-type and mutant COL4A3 amino acid sequences was performed using SignalP 4.1, PrediSi, and Signal-3L 2.0 (http://www.cbs.dtu.dk/services/SignalP/, http://www.predisi.de/, http://www.csbio.sjtu.edu.cn/bioinf/Signal-3L/, last accessed October 2016)

Potential pathogenicity of the candidate *COL4A5* missense variant was assessed using: SIFT, MutationTaster2, PolyPhen-2, MutationAssessor, FATHMM, Likelihood Ratio Test (LRT), Condel, Provean, and CADD [13–21].

### Expression vector preparation

For the functional characterization of the candidate branch-site mutation in *COL4A5*, the relevant genomic DNA region was cloned in the hybrid alpha-globin-fibronectin minigene plasmid (modified pBS-KS), as previously described [22]. In particular, a 543-bp fragment of *COL4A5* (including the entire exon 29 and flanking intronic regions) was PCR amplified from the patient’s genomic DNA using the following primers: COL4A5_ex29_*Nde*I_F 5’-ggaattccatatgACCCTGTTTCCAATCCTTCCA-3’ and COL4A5_ex29_*Nde*I_R 5’-ggaattccatatgGCCGGGCCATGATTTTATT-3’ (lowercase letters indicate nucleotides added to the primers to introduce the *Nde*I restriction site) and cloned into the modified pBS-KS vector.

For the functional characterization of the COL4A3 signal-peptide deletion, the genomic DNA region coding for the predicted signal peptide was PCR amplified from the patient’s genomic DNA (with primers including *Xho*I and *Kpn*I restriction sites: COL4A3_SP_F 5’-ggcctcgaGGTGGCCTGAGAGCCTGA-3’ and COL4A3_SP_R 5’-agaggtaccTGGAGGAGGGATGGAAGTG-3’) and cloned in-frame upstream of the EGFP (Enhanced Green Fluorescent Protein) coding region in the pEGFP-N1 plasmid (Clontech, Mountain View, CA).

Recombinant plasmids were extracted with the PureYield Plasmid Midiprep System (Promega), and verified by sequencing.

### Cell cultures and transfection experiments

HEK293 cells were cultured in Dulbecco's Modified Eagle medium containing 2 mM L-glutamine, 10% fetal bovine serum and antibiotics (100 U/ml penicillin and 100 μg/ml streptomycin; Euroclone, Wetherby, UK) and grown at 37°C in a humidified atmosphere of 5% CO_2_ and 95% air, according to standard procedures.

For splicing assays on *COL4A5* exon 29, an equal number of cells (3x10^5^) were transiently transfected with 1 μg of either the wild-type or the mutant recombinant vector using the JetPRIME reagent (Euroclone), as described by the manufacturer.

Similarly, for localization analyses, 2.5x10^5^ HEK293 cells were seeded on 22x22 mm glass coverslips and transfected with 1 μg of either the wild-type (COL4A3-SP-wt-hybEGFP) or the deleted (COL4A3-SP-del-hybEGFP) recombinant vector. As positive control, the empty pEGFP-N1 plasmid, expressing a soluble EGFP, was used. Cells were fixed 24 hours after transfection using 4% paraformaldehyde, permeabilized, and mounted with ProLong Diamond Antifade Mountant with DAPI (Molecular Probes, Eugene, OR). Confocal images were acquired using a 60x UPLSAPO oil-immersion objective (N.A. 1.35, Olympus, Shinjuku, Tokyo, Japan) with an Olympus FluoView FV1000 confocal microscope at a resolution of 1 airy unit. The fluorescence resulting from the sequential excitation at 405 (Diode laser) and 488 (Argon ion laser) nm was collected with 425–475 (for DAPI) and 500–600 (for EGFP) nm band-pass filters. Identical gain, offset, exposure, and laser-power settings were applied to all acquisitions.

### Splicing analyses

**Fig 2:**
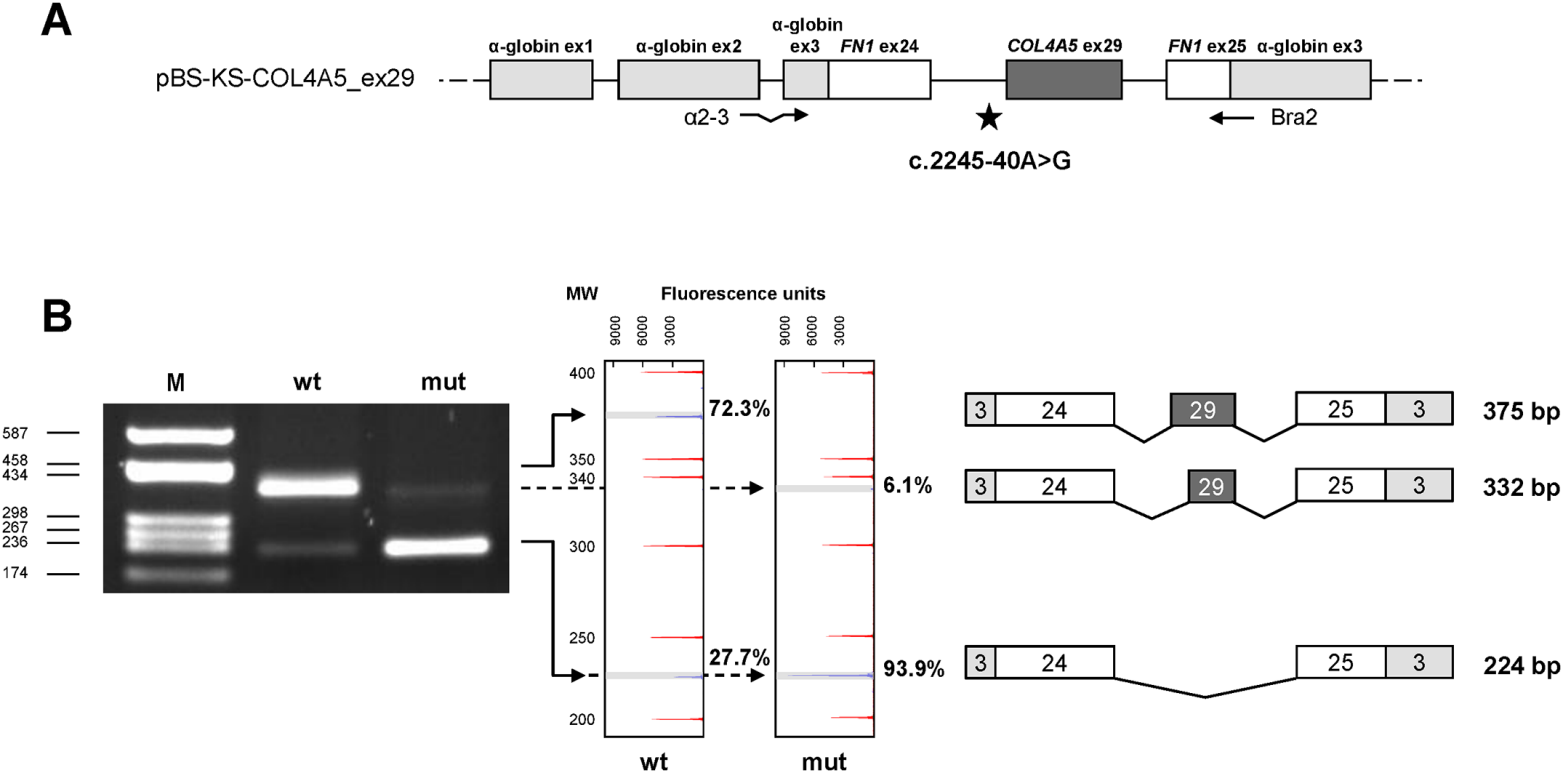
*In-vitro* analysis of the impact of c.2245-40A>G variant on *COL4A5* pre-mRNA splicing. (A) Schematic representation of the hybrid pBS-KS-COL4A5_ex29 minigene where α-globin exons are represented by light grey boxes, fibronectin (*FN1*) exons by white boxes, whereas introns are shown as black lines (not to scale). Exon 29 of *COL4A5* is represented by a dark grey box. The c.2245-40A>G mutation in intron 28 is indicated by a star. Primers used in RT-PCR assays are also indicated. (B) On the left, agarose gel (2%) electrophoresis of RT-PCR products obtained from RNA of HEK293 cells transfected with the wild-type (wt) or mutant (mut) minigene vector. M: molecular weight marker (pUC9-*Hae*III). In the middle, GeneMapper windows show fluorescence peaks corresponding to the molecular species amplified by RT-PCR. Grey shaded peaks correspond to the RT-PCR-labeled products, whose relative quantitation is reported on the right of the panel (%). Unshaded peaks represent the size standard (ROX-500 HD). The *x* axis indicates fluorescence units. On the right, schematic representation of the splicing products, as verified by Sanger sequencing. The length of each fragment is shown.

Total RNA was isolated from cells 24 hours after transfection, using the EuroGold TriFast reagent (Euroclone). Random hexamers and the ImProm-II Reverse Transcriptase (Promega) were used to perform first-strand cDNA synthesis, starting from 500 ng of total RNA, according to the manufacturer's instructions. Of a total of 20 μL of the RT reaction, 1 μL was used as template for amplifications, using primers annealing to the flanking α-globin/*FN1* exonic sequences (α2–3 and Bra2 primers; Fig 2). RT-PCRs were performed under standard conditions using the GoTaq DNA Polymerase (Promega) on a Mastercycler EPgradient (Eppendorf, Hamburg, Germany).

Competitive-fluorescent RT-PCRs were performed on RNA from transfected cells, using the same oligonucleotide pair adopted for splicing assays, with the reverse Bra2 primer labelled with 6-FAM. Amplified fragments were separated on an ABI-3500DX sequencer and quantitated by the GeneMapper v4.0 software (Applied Biosystems). The sum of all fluorescence peak areas in a single run was set equal to 100%, and the relative quantity of each transcript expressed as a fraction of the total.

## Results

### Exome sequencing identified potentially pathogenic AS variants

WES, performed on one affected individual from each of the three families under analysis (P1-P3, Table 1 and Fig 1), yielded on average 7.7 Gb high-quality sequence data/exome, with 99.6% target coverage and a 90X mean coverage depth (S2 Table). We focused data analysis on known Alport genes only, and verified that all exons of *COL4A3*, *COL4A4*, and *COL4A5* were adequately covered by WES (S1 Fig). The less covered exon was *COL4A3* exon 1, with a mean coverage of 31X, 16X, and 42X in P1, P2, and P3, respectively. Specific analysis of rare variants (MAF≤0.01) within AS genes, including all intronic variants, identified a candidate pathogenic variant in each patient (S3 Table).

P1 is a 47-year-old woman with history of hematuria and slight proteinuria. Diagnosis of AS was made on the basis of kidney biopsy. She referred that several relatives on her mother’s side had renal problems and/or died for end-stage renal disease, although her mother does not show an overt renal phenotype, apart from microhematuria. The proband’s daughter (currently 21 y.o.) has hematuria, developed proteinuria at age 8, which is progressively worsening, and also presented with sensorineural hearing loss (SHL) with onset in the first decade. WES data analysis detected a novel heterozygous A-to-G transition (NM_000495.4:c.2245-40A>G) within *COL4A5* intron 28, 40 nucleotides upstream of exon 29. The variant is present in P1’s mother and daughter, whereas it is absent in her unaffected siblings (Fig 1A). The peculiar location of this variant suggested it might affect the branch-point sequence, a conserved signal important for spliceosome assembly and lariat formation.

The male P2 proband, who is currently 20 years old, started to show microhematuria and episodes of macrohematuria with proteinuria at age 3; renal biopsy evidenced thinning, thickening, and splitting of the GBM, supporting a diagnosis of AS. Proteinuria has progressively worsened, with current ratio of proteinuria/creatinuria >2. He has chronic kidney disease with glomerular filtration rate (GFR)<50 ml/min. He developed SHL at age 10. No ocular signs are present. The proband’s mother presented with urinary anomalies (microhematuria and proteinuria) and SHL, but normal renal function. The proband’s grandmother only showed microhematuria.

WES data analysis identified a novel *COL4A5* missense variant within exon 33 (NM_000495.4:c.2822G>A), causing the substitution of glycine 941 with an aspartic acid (p.Gly941Asp). The variant is present in the hemizygous state in the proband as well as in the affected male cousin (IV1), and in the heterozygous state in all affected female relatives (Fig 1B). In addition, the identified missense variant is absent both in an in-house database of ~3,500 ethnically-matched control exomes and in the ExAC browser (http://exac.broadinstitute.org/, last accessed October 2016), suggesting that it likely represents a private mutation. The NM_000495.4:c.2822G>A variant affects an evolutionary-conserved residue and is predicted to be damaging by 10 out of 10 commonly used programs to predict the deleteriousness of an amino acid substitution (S2 Fig). Indeed, glycine substitutions within the repetitive triplet sequence (Gly)-X-Y of the collagenous domain represent one of the most common type of pathogenic variant found in AS patients, as they are suspected to introduce kinks in the molecule, thus interfering with the proper folding of the collagen triple helix [2]. Notably, a different missense mutation affecting the same amino acid, c.2821G>T (p.Gly491Cys), was previously reported [23,24].

P3 is a 25-year-old woman with family history of isolated microhematuria. She first presented with microhematuria at age 2 and subsequently developed proteinuria at 7, which has been progressively worsening. Renal biopsy was diagnostic for AS. At the end of the first decade, she developed SHL and she is wearing bilateral hearing aids. At 21, routine ophthalmic evaluation evidenced a slight maculopathy, characterized by macular flecks, which has remained stable ever since.

WES showed that P3 carries a homozygous deletion in *COL4A3* on chromosome 2 (NM_000091.4:c.30_53del:p.Val11_Leu18del or NM_000091.4:c.40_63del24:p.Leu14_Leu21del). The variation was found in the heterozygous state in the mother (II5), who suffers from micro-hematuria (Fig 1C). No overt consanguinity was reported, even though a run of homozygosity, spanning >18 Mb from gene *EPHA4* to gene *PRR21*, was found on chromosome 2. We ruled out the presence of a large deletion on the second allele in the proband by quantitative real-time PCR (data not shown), but we could not exclude a partial uniparental disomy. This 24-bp in-frame deletion in exon 1 is annotated as a rare low-quality variant (minor allele frequency, MAF = 0.047%) in the ExAC browser (the site is covered in <80% of analyzed individuals) and is present in dbSNP147 with two different accession numbers (rs774798108 or rs876657397). This deletion has been previously and repeatedly reported in AS patients [25–28]. The 24-bp deletion would eliminate 8 amino acids from the signal peptide, possibly altering *COL4A3* protein secretion, as predicted by three different programs (S3 Table).

### The c.2245-40A>G variant in *COL4A5* affects exon 29 splicing by abolishing the branch site

The *COL4A5* intronic variant identified in Family 1 (NM_000495.4:c.2245-40A>G) occurs in a region compatible with exon 29 branch site. According to in-silico predictions performed with Human Splicing Finder, the wild-type residue corresponds to the best-scoring branch-point site in the region comprised between nucleotides -20 to -40 upstream of exon 29 acceptor splice site, although it does not reach the software threshold for significance (i.e. 67). However, the c.2245-40A>G substitution significantly decreases the score of the potential branch site, from 59.27 to 29.64, suggesting that this variant might impact on exon 29 recognition.

Given the unavailability of a suitable biological specimen from the patient to extract RNA, *COL4A5* exon 29, with the surrounding intronic sequences, was cloned, either in the wild-type or in the mutant version, into a pBS-KS_modified hybrid minigene vector (Fig 2A). The obtained constructs (pBS-KS-*COL4A5*_ex29_wt and pBS-KS-*COL4A5*_ex29_mut) were transiently transfected into human renal HEK293 cells, and *COL4A5* splicing was analyzed by reverse-transcription (RT)-PCR. All amplified fragments were then subjected to direct sequencing to characterize aberrant splicing events. Furthermore, relative quantitation of all splicing isoforms was performed by competitive-fluorescent RT-PCRs. Transfection with the mutant vector originated two aberrant products: the most abundant (93.9%) resulting from the skipping of exon 29, and the other one (6.1%) resulting from the inclusion of a shorter exon 29, caused by the activation of an exonic cryptic 3' acceptor site (Fig 2B). No residual wild-type splicing was detected. Exon 29 skipping was also present in a fraction (27.7%) of transcripts derived from the wild-type construct. Both the skipping of exon 29 and the inclusion of the shorter exon 29 are predicted to cause frameshifts leading to the introduction of a premature stop after 767 or 776 amino acids, respectively.

### Phenotypic variability within Family 1 correlates with skewed inactivation of the mutant allele

Since the three female carriers of the branch-point NM_000495.4:c.2245-40A>G variant showed different severity of phenotypic manifestations (Fig 1A), we investigated the possibility that these differences might be due to skewed X-inactivation. Hence, to evaluate the methylation status of the two alleles, we performed a methylation-sensitive restriction-enzyme assay on DNA extracted from patients’ blood and then we discriminated the wild-type from the mutant allele using a polymorphic marker cosegregating with *COL4A5*, for which the tested females were heterozygous (Supporting Methods). The results showed a skewed inactivation (91%) of wild-type *COL4A5* allele in IV1, a balanced inactivation of both alleles in III3 (45 vs 55%), and a greater inactivation (89%) of the mutant *COL4A5* allele in II2 (S3 Fig), nicely correlating with the progressively milder phenotypic manifestations.

### The 24-bp deletion in *COL4A3* affects the signal peptide

**Fig 3:**
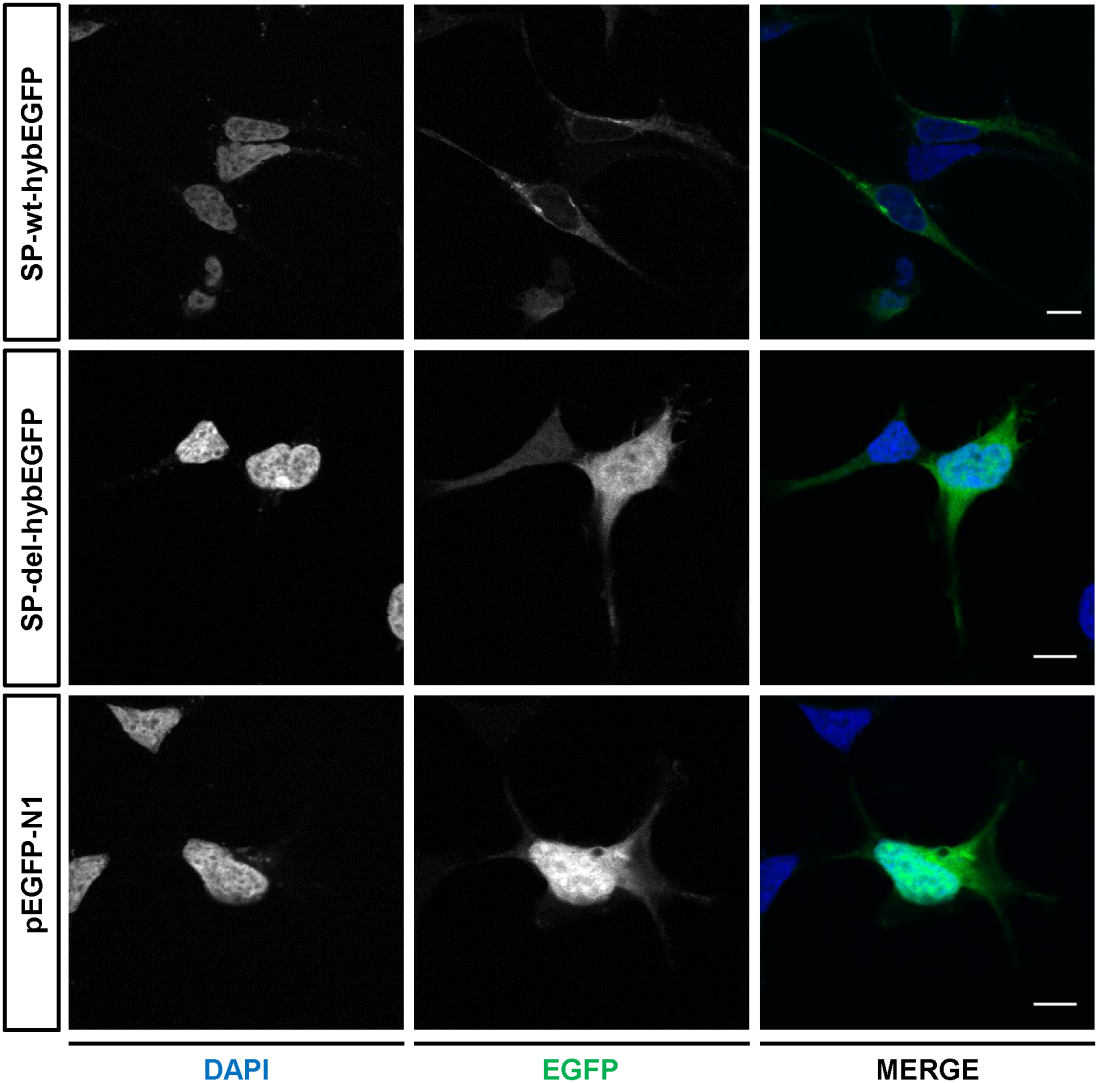
Functional characterization of the signal peptide deletion in COL4A3. Single confocal sections of HEK293 cells expressing EGFP N-terminus fused either with the entire COL4A3 signal peptide (SP-wt-hybEGFP, top panels) or the 8-amino-acid deleted signal peptide (SP-del-hybEGFP, middle panels). Positive control cells, expressing a soluble EGFP (pEGFP-N1) are also shown (bottom panels). DAPI, 4',6-diamidino-2-phenylindole; EGFP, Enhanced green fluorescent protein. Scale bar: 10 μm.

To demonstrate that the NM_000091.4:c.30_53del (or c.40_63del) variant alters the physiologic COL4A3 signal peptide, we performed localization assays in HEK293 cells using a hybrid fluorescent reporter protein (hybEGFP, Enhanced Green Fluorescent Protein) containing at the N-terminus either the complete COL4A3 signal peptide (amino acids 1–29) or the partially deleted localization signal (Val11_Leu18del). Our results show a clear difference in hybEGFP subcellular localization in the presence of the deleted COL4A3 signal peptide compared to the wild type (Fig 3). In particular, hybEGFP fused with the mutant peptide lacking 8 amino acids displays a diffuse and uniform localization in the cytoplasm and in the nucleus, similarly to the soluble EGFP (Fig 3). On the contrary, COL4A3-SP-wt-hybEGFP shows a more punctate distribution within the cytoplasm, compatible with localization along the secretory pathway. Co-localization assays using either a marker of the endoplasmic reticulum (the calnexin protein), or a marker of the Golgi apparatus (Trans-Golgi Network protein 38, TGN38) seem to support this hypothesis (S4 Fig).

## Discussion

Here we used WES -together with functional studies- to provide three AS families with a molecular diagnosis after years of inconclusive results, highlighting non-obvious pathogenic variants that escaped previous screenings.

The identification of the *COL4A5* branch-point variant represented a diagnostic challenge primarily for its location. Indeed, in routine molecular testing (Sanger sequencing-based) there is no clear guideline for the intronic regions to include in the screening [29]. Consensus splice sites and surrounding nucleotides (+1 to +6 and –10 to –1 bp) are usually analyzed, whereas branch points often are not. The same holds true when NGS approaches are applied in diagnostic settings [30–32]. Hence, disease-causing variants residing in other intronic locations (including the branch point) may go undetected, unless an analysis on patient’s RNA is performed [33,34]. In addition, branch points are difficult to predict on the basis of the nucleotide sequence alone, due to weakly-conserved consensus sequences [35], and to the absence, until very recently, of experimentally-generated genome-wide datasets of human branch sites [36].

Branch-point selection is an early step in the splicing process that defines the 3’ acceptor site and leads to inclusion of the downstream exon in the mature mRNA [37]. Mutations that affect the branch-point sequence, particularly the branch-point adenine or the common uridine 2 nucleotides upstream of it (UNA motif), can result in exon skipping or other aberrant splicing events [38]. Here, we identified a *COL4A5* intronic variant in Family 1 (NM_000495.4:c.2245-40A>G) that likely represents the branch-point nucleotide required for lariat formation and correct inclusion of exon 29. Although prediction programs failed to recognized it as a reliable branch site, its location is compatible with the distance of the branch-point adenine from the 3′ splice acceptor, which, for the 90% of branch points, is within 39 nucleotides [36]. Moreover, the affected residue (hg19, chrX:107849932) is conserved across vertebrates (Phastcons score = 1). Finally and most convincingly, we showed abnormal *COL4A5* mRNA splicing in the presence of the NM_000495.4:c.2245-40A>G substitution, using an in-vitro splicing assay. To our knowledge, this is the first report of a branch-site mutation in AS genes, and adds to the relatively small number of branch point-sequence lesions identified as genetic causes of human diseases [34,38–40]. Indeed, only five ClinVar SNPs deleting branch points are annotated in OMIM [41].

On the other hand, the identification of the 40_63del variant within exon 1 of *COL4A3* in Family 3 represented a diagnostic challenge mainly for technical reasons. *COL4A3* exon 1 is extremely GC-rich (77%), and has been reported to be difficult to screen with benchtop NGS sequencers such as the Ion PGM [8]. Actually, it was not amplified and analyzed in previous *COL4A3*-targeted NGS analysis on our patient. In our WES data, *COL4A3* exon 1 was the least covered exon among the three type IV collagen genes (S1 Fig). A second issue relating to this specific mutation is its correct mapping and nomenclature. Due to the repetitive nature of the affected sequence, the deletion could be aligned to the reference genome sequence in two ways, leading to different naming: NM_000091.4:c.30_53del:p.Val11_Leu18del or NM_000091.4:c.40_63del:p.Leu14_Leu21del (S5 Fig). Each of these annotations is associated with a different dbSNP 147 accession number (rs774798108 or rs876657397), although commonly used genome browsers (UCSC, https://genome.ucsc.edu/, and ENSEMBL, http://www.ensembl.org/, last accessed 30 December 2016) only report the rs774798108 variant, which lacks clinical annotation; conversely, rs876657397 is annotated as a pathogenic allele (OMIM: 120070.0011; ClinVar: 192299). We hypothesize that the first annotation derives from automated short-read alignment of NGS data, whereas the second possibly resulted from inspection of electropherograms obtained by Sanger sequencing, and conforms to the Human Genome Variation Society (HGVS) recommendations for the description of sequence variants (http://varnomen.hgvs.org). Indeed, over the years, the 40_63del variant was repeatedly reported in AS patients of different ethnic origins. It was first described in the heterozygous state in an Italian patient with history of microhematuria and mild proteinuria [25]. Subsequently, it was found in one Spanish patient [26] and four Chinese patients [27] with autosomal recessive AS: in four of them, the mutation was present in compound heterozygosity with other pathogenic mutations [26,27], whereas in the fifth patient it was present in the homozygote state, despite no reported consanguinity [27]. More recently, the same in-frame deletion was identified as a founder mutation in Ashkenazi Jews, with an estimated carrier frequency of 1:183 [28]. In the heterozygous state, the 40_63del mutation in *COL4A3* seems to cause little or no renal dysfunction, whereas in the homozygous state or combined, on the other allele, with another loss-of-function mutation, it results in severe AS with hearing loss and, as in the case of our proband, ocular anomalies.

From a purely genetic point of view, there is little doubt on the pathogenicity of this variant; however, at the moment, automated alignment and annotation could lead to miss its correct identification as pathogenic. We hence suggest to make uniform and to update the nomenclature for this variant, possibly merging the two annotations, to avoid misleading interpretations of its clinical relevance. In addition, we provide for the first time a functional characterization of this mutation demonstrating that it alters the COL4A3 signal peptide, thus adding a further level of evidence for pathogenicity. Due to the recurrence of this specific variant in different populations, it would be important to screen specifically *COL4A3* exon1 whenever a diagnosis of autosomal recessive AS and/or familiarity for isolated microhematuria is suspected.

In conclusion, we demonstrated how a diligent application of exome-sequencing data analysis combined with an accurate experimental validation are critical to solve elusive cases in the molecular genetic diagnosis of AS. This concept is valid for most human genetic diseases and is becoming increasingly important in the P4 (Personalized, Predictive, Preventive, Participatory) medicine era.

## Supporting information

S1 Table Summary of previous genetic screenings. (DOCX)

S2 Table Whole-exome sequencing (WES) statistics. (DOCX)

S3 Table WES variants within known Alport syndrome genes. (XLSX)

S4 Table Signal peptide prediction of wild-type and mutant COL4A3 amino acid sequences. (DOCX)

S1 Fig Exon-based coverage statistics of Alport syndrome genes from WES data. (DOCX)

S2 Fig *In-silico* analyses of the novel COL4A5 p.Gly941Asp missense variant identified in Family 2. (DOCX)

S3 Fig X-inactivation analysis of Family 1. (DOCX)

S4 Fig Co-localization studies of hybEGFP with markers of the secretory pathway. (DOCX)

S5 Fig Misleading annotation of 24-bp deletion variant within *COL4A3* exon1 in public databases. (DOCX)

S1 File Supplementary methods. (DOCX)
