# Supplementary material for: Alport syndrome cold cases: Missing mutations identified by exome sequencing and functional analysis
Source: PLoS One. 2017 Jun 1;12(6):e0178630. doi: 10.1371/journal.pone.0178630 (PMC5453569; doi:10.1371/journal.pone.0178630)
Supplement: S1 Table — (DOCX) [file pone.0178630.s001.docx]

**S1 Table. Summary of previous genetic screenings**

| **Patient** | **SSCP** | **DHPLC** | **454 Junior Platform (NGS)** | |
| --- | --- | --- | --- | --- |
|  |  |  | **Gene** | **Missing exons** |
| **P1** | *COL4A5* | n.a. | *COL4A5* | 2, 23 |
|  | n.a. | *COL4A4* | *COL4A4* | 25, 28, 29, 41 |
|  | n.a. | n.a. | *COL4A3* | 28, 46 |
| **P2** | *COL4A5* | n.a. | *COL4A5* | 11,12, 14, 15, 18, 33 |
|  | n.a. | *COL4A4* | *COL4A4* | 16, 18, 25, 30 |
|  | n.a. | n.a. | *COL4A3* | 13, 37, 44 |
| **P3** | *COL4A5* | n.a. | *COL4A5* | 1, 4, 23, 33 |
|  | n.a. | n.a. | *COL4A4* | 25, 29, 32, 48 |
|  | n.a. | n.a. | *COL4A3* | 44 |

SSCP, Single-Strand Conformation Polymorphism; DHPLC, Denaturing High Performance Liquid Chromatography; n.a., not analyzed.
