# Supplementary material for: Alport syndrome cold cases: Missing mutations identified by exome sequencing and functional analysis
Source: PLoS One. 2017 Jun 1;12(6):e0178630. doi: 10.1371/journal.pone.0178630 (PMC5453569; doi:10.1371/journal.pone.0178630)
Supplement: S2 Table — (DOCX) [file pone.0178630.s002.docx]

**S2 Table. Whole-Exome Sequencing (WES) statistics**

|  | **P1** | **P2** | **P3** | **Mean** |
| --- | --- | --- | --- | --- |
| **Total nr of HQ bases (Gb)** | 9.69 | 6.09 | 7.47 | 7.75 |
| **Total nr of HQ bases on target (Gb)** | 4.47 | 2.62 | 3.08 | 3.39 |
| **Total aligned reads (M)** | 85.04 | 51.74 | 57.98 | 64.91 |
| **Reads mapped to target±150bp** | 57,446,408 | 31,928,821 | 37,028,299 | 42,134,509 |
| **Target Size**^1^ **(Mb)** | 45.3 | 45.3 | 45.3 | 45.3 |
| **Mean Coverage** | 121 | 68.8 | 81.6 | 90.5 |
| **Covered 1x (%)** | 99.7 | 99.5 | 99.7 | 99.6 |
| **Covered 10x (%)** | 94.6 | 92.2 | 95.3 | 94.0 |
| **Covered 20x (%)** | 89.4 | 82.4 | 88.2 | 86.7 |
| **Covered 30x (%)** | 84.2 | 71.7 | 79.3 | 78.4 |

M, million; HQ, high quality

^1^Nextera Rapid Capture Exome v1.2
