# Supplementary material for: Alport syndrome cold cases: Missing mutations identified by exome sequencing and functional analysis
Source: PLoS One. 2017 Jun 1;12(6):e0178630. doi: 10.1371/journal.pone.0178630 (PMC5453569; doi:10.1371/journal.pone.0178630)
Supplement: S4 Table — (DOCX) [file pone.0178630.s004.docx]

**S4 Table. Signal peptide prediction of wild-type and mutant COL4A3 amino acid sequences**

| *COL4A3* sequence | SignalP 4.1^1^ | | | PrediSi^2^ | | | | Signal-3L^3^ | |
| --- | --- | --- | --- | --- | --- | --- | --- | --- | --- |
|  | **Score** | **Cleavage position** | **Signal Peptide** | **Score** | **Cleavage position** | **Signal Peptide** | **Secreted?** | **Cleavage position** | **Signal Peptide** |
| *Wild type* | 0.79 | 28 | Y | 1.00 | 28 | Y | Y | 26 | Y |
| *24bp-del*  *(Family 3)* | 0.21 | n.c. | N | 0.45 | 22 | N | N | n.c. | N |
| *rs570469692*  *(12-bp del)* | 0.71 | 26 | Y | 1.00 | 26 | Y | Y | 26 | Y |
| *24bp-del*  *(Longo et al. 2002)* | 0.21 | n.c. | N | 0.45 | 22 | N | N | n.c. | N |

n.c. not calculated

1 SignalP 4.1, http://www.cbs.dtu.dk/services/SignalP/

2 PREDIction of SIgnal peptides, http://www.predisi.de/

3 Signal-3L 2.0, http://www.csbio.sjtu.edu.cn/bioinf/Signal-3L/
