# Supplementary material for: Alport syndrome cold cases: Missing mutations identified by exome sequencing and functional analysis
Source: PLoS One. 2017 Jun 1;12(6):e0178630. doi: 10.1371/journal.pone.0178630 (PMC5453569; doi:10.1371/journal.pone.0178630)
Supplement: S1 Fig — (DOCX) [file pone.0178630.s005.docx]

**S1 Fig. Exon-based coverage statistics of Alport syndrome genes from WES data.**

**A.**

|  | **P1** | | | **P2** | | | **P3** | | |
| --- | --- | --- | --- | --- | --- | --- | --- | --- | --- |
|  | **min** | **mean** | **max** | **min** | **mean** | **max** | **min** | **mean** | **max** |
| ***COL4A5*** | 105 | 205 | 326 | 33 | 63 | 94 | 64 | 133 | 212 |
| ***COL4A4*** | 63 | 186 | 357 | 41 | 109 | 210 | 40 | 118 | 215 |
| ***COL4A3*** | 31 | 207 | 435 | 16 | 124 | 251 | 43 | 132 | 288 |

**B.**
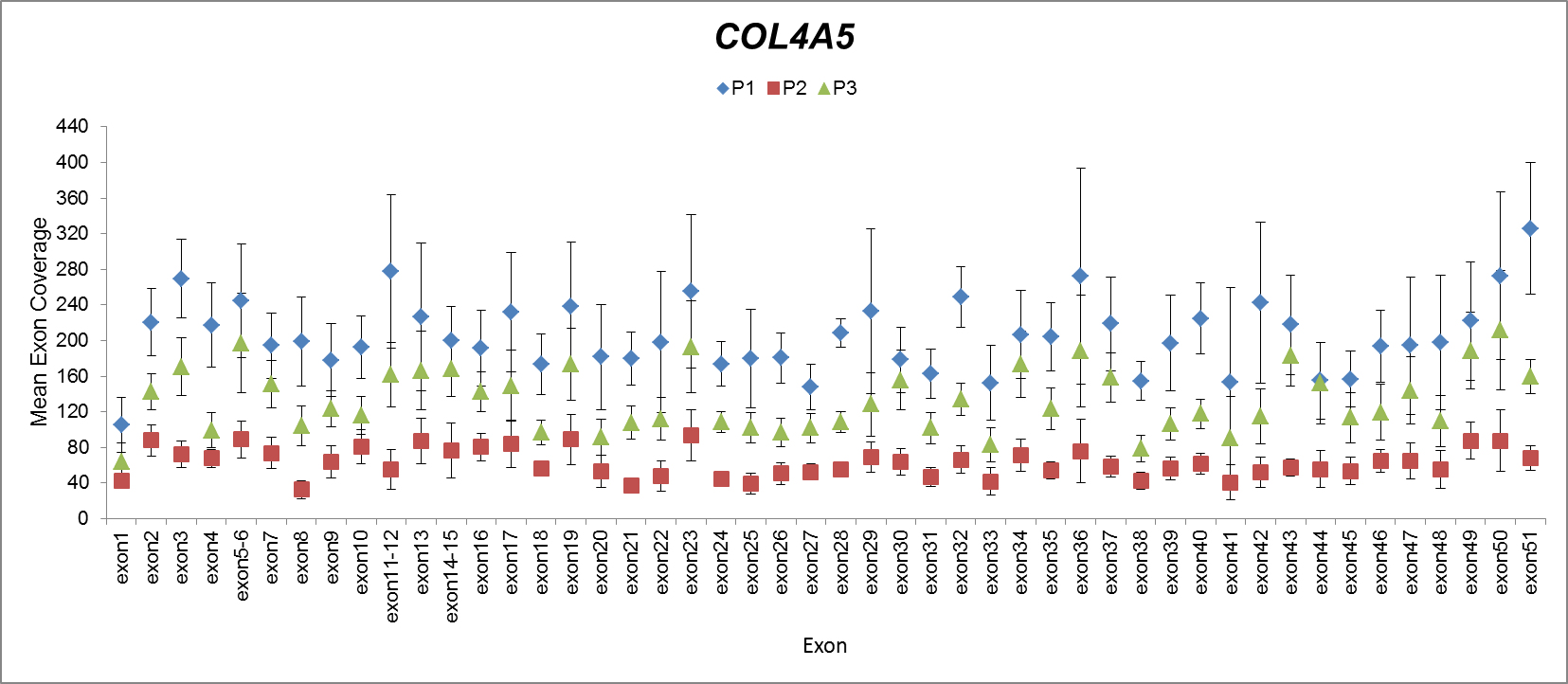


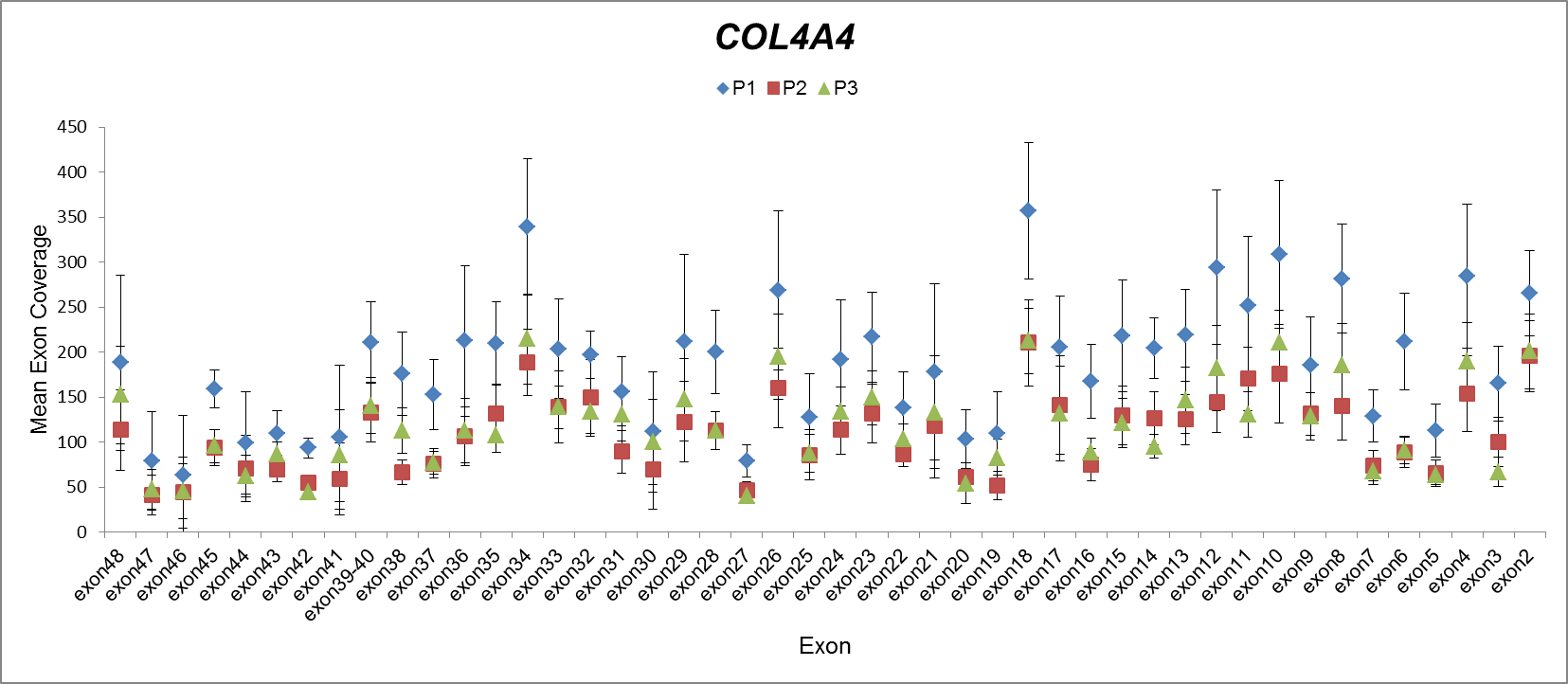


**C.**


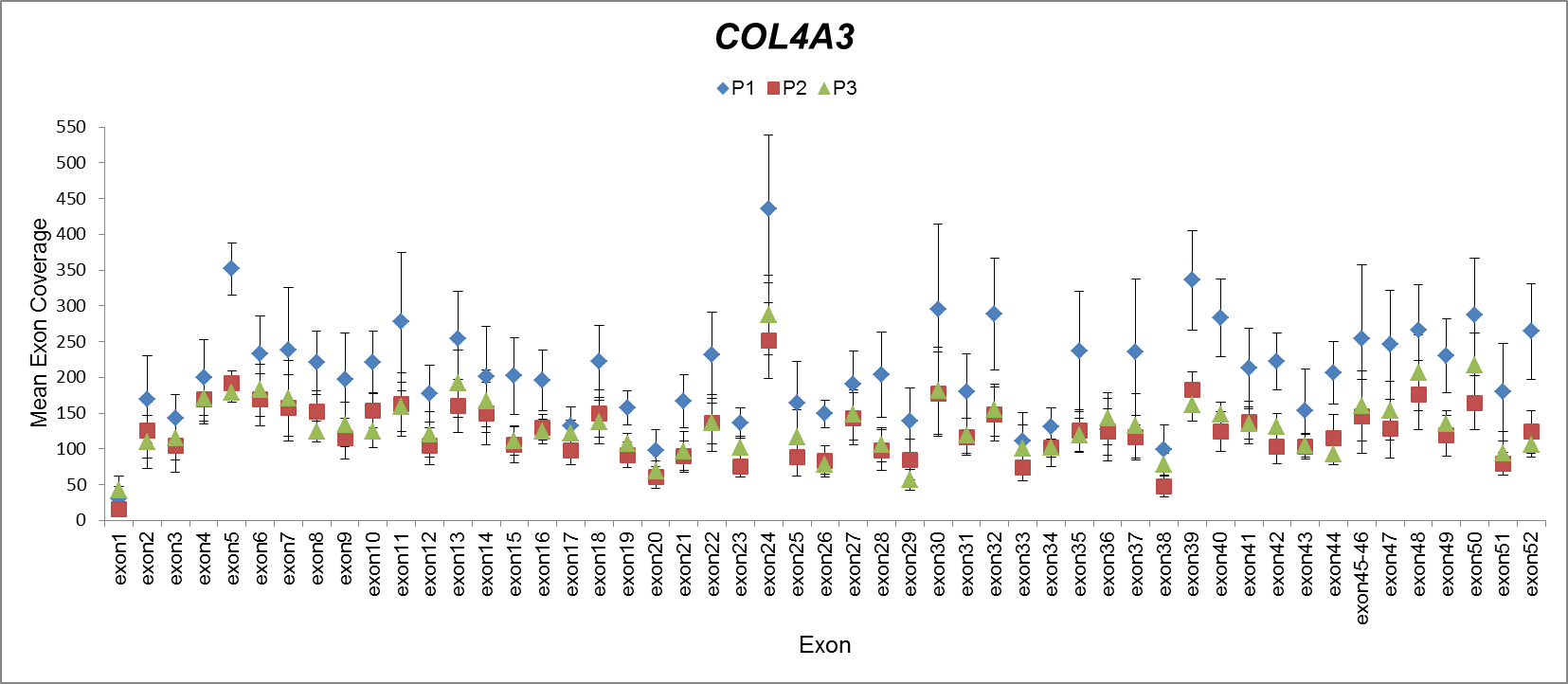


**D.**
