# Supplementary material for: Alport syndrome cold cases: Missing mutations identified by exome sequencing and functional analysis
Source: PLoS One. 2017 Jun 1;12(6):e0178630. doi: 10.1371/journal.pone.0178630 (PMC5453569; doi:10.1371/journal.pone.0178630)
Supplement: S2 Fig — (DOCX) [file pone.0178630.s006.docx]

**A**


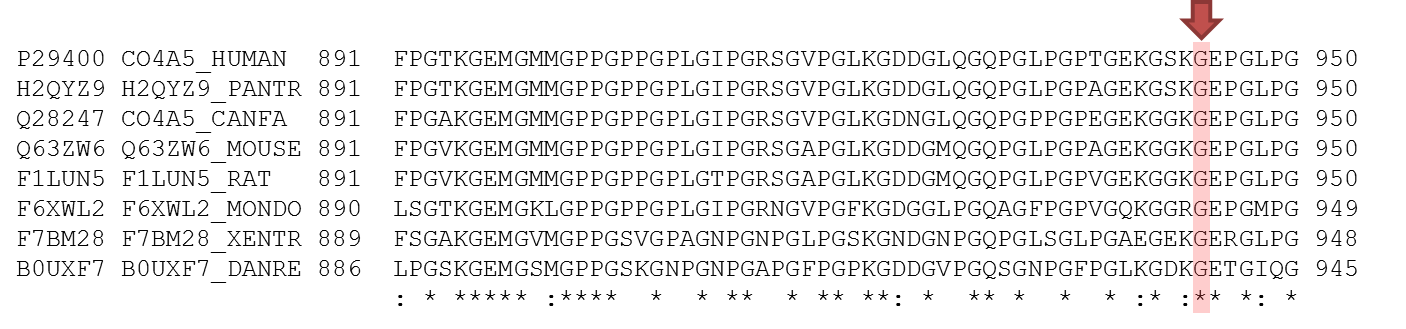


**B**

|  | ***COL4A5* Missense variant** |
| --- | --- |
| **Predictor** | **p.Gly941Asp** |
| **SIFT (score)** | D (0.00) |
| **Polyphen 2 HumDiv (score)** | PD (1.00) |
| **Polyphen 2 HumVar (score)** | PD (1.00) |
| **LRT** | D |
| **MutationTaster** | Disease causing |
| **Mutation Assessor^1^** | FIS H |
| **FATHMM (weighted score)** | D (-6.28) |
| **Condel (score)** | D (0.733) |
| **Provean (score)** | D (-8.374) |
| **CADD** | scaled C-score 20.7 |

**S2 Fig. *In silico* analyses of the novel *COL4A5* p.Gly941Asp missense variant identified in Family 2.**

**A. Protein sequence alignments of COL4A5 orthologs in the region surrounding the mutant residue (p.Gly941).** Protein sequences were retrieved from UniProt, and alignments were generated with Clustal Omega. The amino acid residue affected by the mutation is boxed in red and indicated by an arrow. Identical amino acid are marked by an asterisk, while partially conserved residues are indicated by a colon.

**B. Pathogenicity prediction of the p.Gly941Asp missense variant with 10 commonly-used software.** LRT, Likelihood Ratio Test; D, deleterious; PD, possibly damaging; FIS, Functional Impact Score; CADD, Combined Annotation Dependent Depletion).

FIS score is ranked as: high (H), medium (M) for predicted functional variants and low (L) for predicted non-functional variants.

CADD Scaled C-score represents the PHRED-like [-10*log10(rank/total)] score, ranking a variant relative to all possible substitutions of the human genome (8.6x10^9^). A scaled C-score of greater of equal 10 indicates that these are predicted to be the 10% most deleterious substitutions that you can do to the human genome, a score of greater or equal 20 indicates the 1% most deleterious and so on.
