# Supplementary material for: Alport syndrome cold cases: Missing mutations identified by exome sequencing and functional analysis
Source: PLoS One. 2017 Jun 1;12(6):e0178630. doi: 10.1371/journal.pone.0178630 (PMC5453569; doi:10.1371/journal.pone.0178630)
Supplement: S3 Fig — (DOCX) [file pone.0178630.s007.docx]

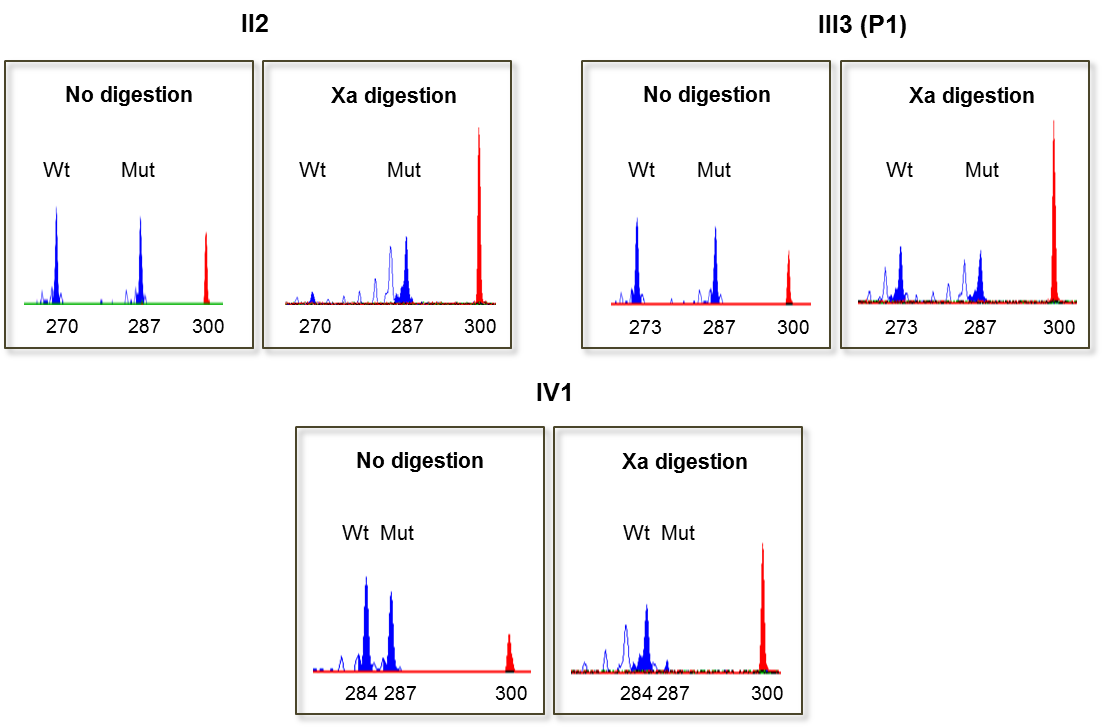


**S3 Fig. X-inactivation analysis of Family 1.**

GeneMapper windows with peaks showing the X-inactivation patterns in the female carriers of the *COL4A5* c.2245-40A>G mutation. Methylation assays were performed on the *AR* CAG polymorphic region. No digestion: undigested genomic DNA; Xa digestion: DNA predigested with the methylation sensitive enzyme *Hpa*II, which only cuts restriction sites on the unmethylated, active X (Xa). The size (bp) of the amplification products for the CAG-repeat region on the two alleles is indicated below the corresponding peak. GeneScan 500 ROX Size Standard is shown in red. Wt: wild-type allele; Mut: mutant allele.
