# Supplementary material for: Alport syndrome cold cases: Missing mutations identified by exome sequencing and functional analysis
Source: PLoS One. 2017 Jun 1;12(6):e0178630. doi: 10.1371/journal.pone.0178630 (PMC5453569; doi:10.1371/journal.pone.0178630)
Supplement: S4 Fig — (DOCX) [file pone.0178630.s008.docx]

**
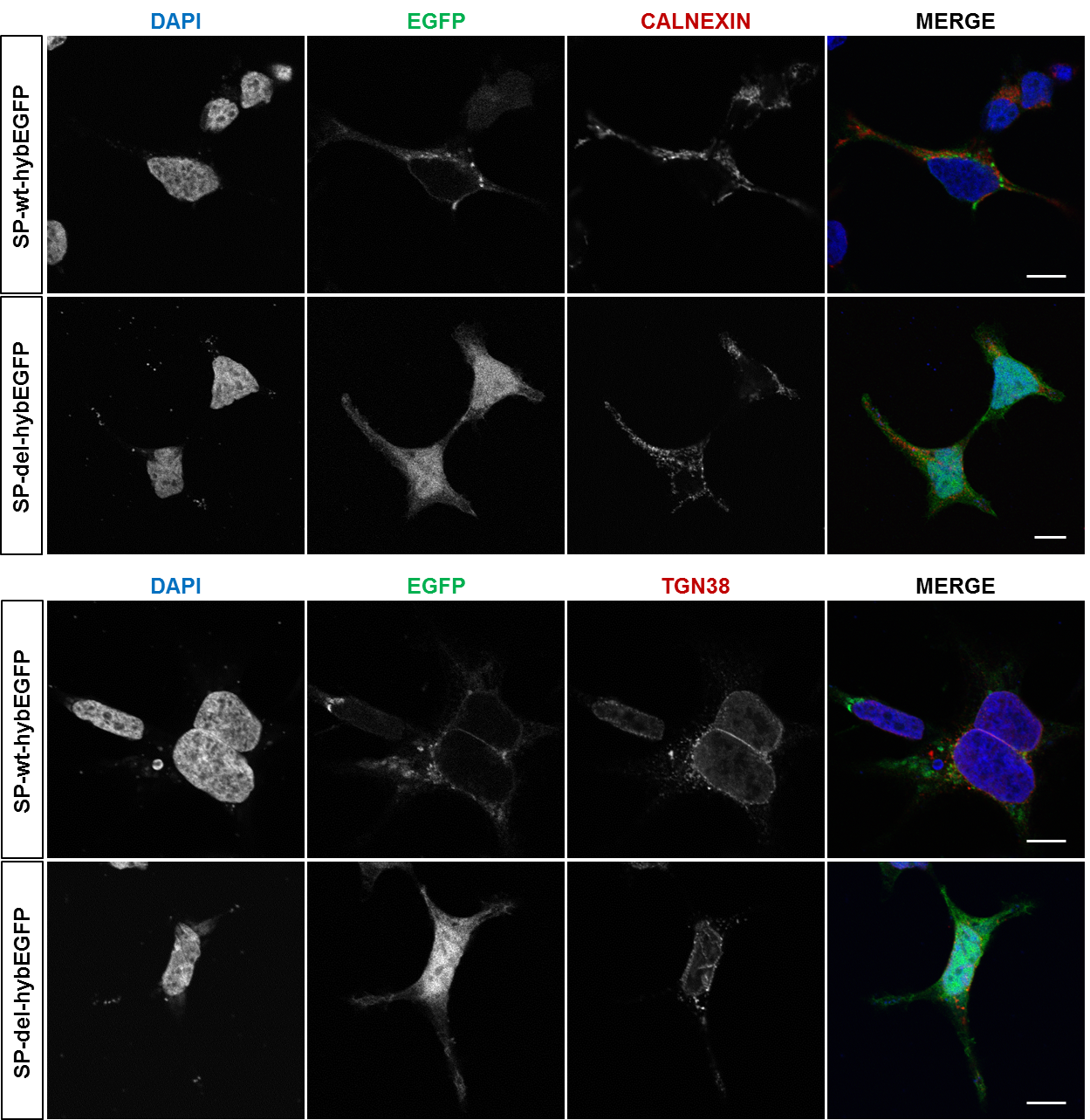
**

**S4 Fig. Co-localization studies of hybEGFP with markers of the secretory pathway.**

Single confocal sections of HEK293 cells expressing COL4A3-SP-wt-hybEGFP or COL4A3-SP-del-hybEGFP and stained for calnexin (endoplamic reticulum transmembrane protein, top panel) or TGN38 (trans-Golgi network protein, bottom panels). Scale bar: 10 μm.
