# Supplementary material for: Alport syndrome cold cases: Missing mutations identified by exome sequencing and functional analysis
Source: PLoS One. 2017 Jun 1;12(6):e0178630. doi: 10.1371/journal.pone.0178630 (PMC5453569; doi:10.1371/journal.pone.0178630)
Supplement: S5 Fig — (DOCX) [file pone.0178630.s009.docx]

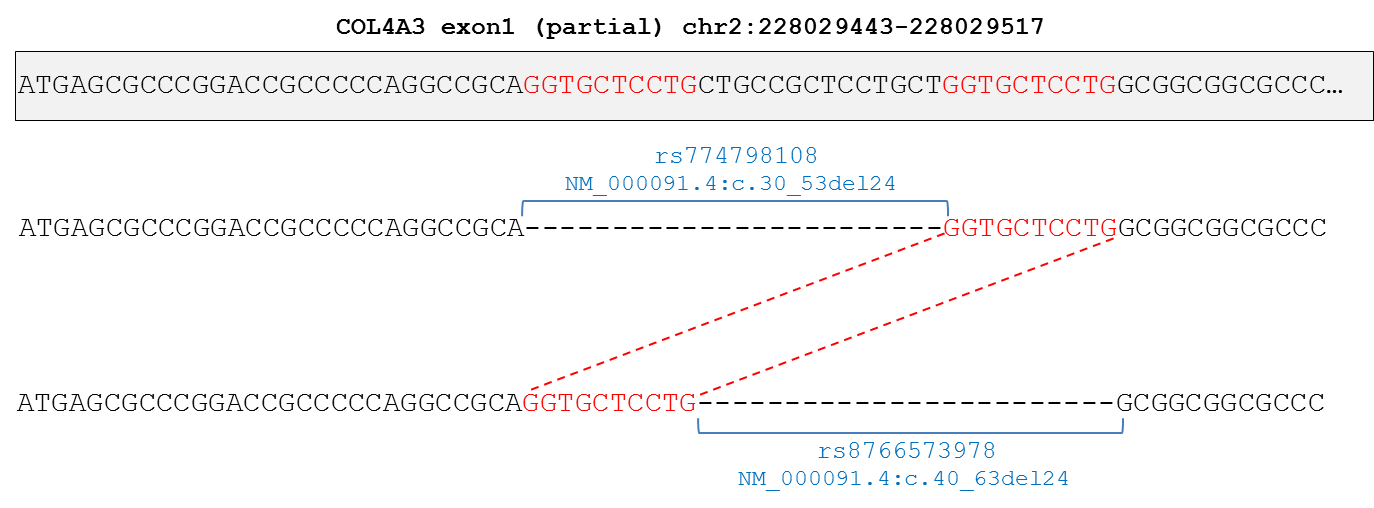
**S5 Fig. Misleading annotation of 24-bp deletion variant within COL4A3 exon1 in public databases.** Sequence carrying the 24-bp deletion can be aligned to the reference genome in two different ways, resulting in different annotations : 1) rs774798108, corresponding to deletion of bases from 228029472 to 228029495 (GGTGCTCCTGCTGCCGCTCCTGCT), and 2) rs876657397 (OMIM: 120070.0011; ClinVar: 192299), corresponding to 228029482_228029505 deletion (CTGCCGCTCCTGCTGGTGCTCCTG). Coordinates refer to the hg19 reference genome.
