# Supplementary material for: Alport syndrome cold cases: Missing mutations identified by exome sequencing and functional analysis
Source: PLoS One. 2017 Jun 1;12(6):e0178630. doi: 10.1371/journal.pone.0178630 (PMC5453569; doi:10.1371/journal.pone.0178630)
Supplement: S1 File — (DOCX) [file pone.0178630.s010.docx]

**Supplementary Methods**

*WES variant annotation and prioritization*

For the prioritization of potentially pathogenic variants, artefacts were filtered out by comparison with in-house sequenced exomes obtained with the same protocol on the same instrument. Common SNVs and indels were excluded by filtering against the 1000 Genome Project, the Exome Variant Server (National Heart, Lung, and Blood Institute Exome Sequencing Project, NHLBI-ESP; 6,500 exomes), and the Exome Aggregation Consortium (ExAC; 60,706 exomes) databases. The threshold for minor allele frequency (MAF) was set to 1%.

*X-inactivation analysis*

Skewing of X-inactivation in female carriers of the *COL4A5* c.2245-40A>G mutation was investigated by analyzing a polymorphic CAG repeat in the promoter region of the Androgen Receptor (*AR*) gene, for which the tested females were heterozygous, by modifying a previously described protocol.^42^ Briefly, 100 ng of genomic DNA was digested overnight at 37°C using a combination of 1 U *Rsa*I and 2.5 U of the methylation-sensitive restriction enzyme *Hpa*II (New England Biolabs, Beverly, MA, USA), as well as 1 U *Rsa*I alone as a negative control, in a total volume of 20 µL. To amplify the polymorphic trinucleotide repeat, an aliquot (5 µL) of the digestion reaction was used as template for PCR with a fluorescein-labeled reverse oligonucleotide (Bodega et al., 2006 and the Go Taq Polymerase (Promega), in 10% DMSO. PCR reactions were separated on an ABI-3500DX sequencer and the peak areas measured by the GeneMapper v4.0 software.

**References**

Bodega B, Bione S, Dalprà L, et al. Influence of intermediate and uninterrupted FMR1 CGG expansions in premature ovarian failure manifestation. Hum Reprod. 2006;21:952-957.

Coene KL, Roepman R, Doherty D, et al. OFD1 is mutated in X-linked Joubert syndrome and interacts with LCA5-encoded lebercilin. Am J Hum Genet. 2009;85:465-481.
